# A note on the wedge reversion antisymmetry operation and 51 types of physical quantities in arbitrary dimensions

**DOI:** 10.1107/S2053273323003303

**Published:** 2023-06-05

**Authors:** Piotr Fabrykiewicz

**Affiliations:** a Institute of Crystallography, RWTH Aachen University, Jägerstraße 17–19, D-52066 Aachen, Germany; b Jülich Centre for Neutron Science at Heinz Maier-Leibnitz Zentrum, Forschungszentrum Jülich GmbH, Lichtenbergstraße 1, D-85747 Garching, Germany; c Faculty of Physics, University of Warsaw, Pasteura 5, PL 02-093 Warsaw, Poland; Columbia University, USA

**Keywords:** multivectors, wedge reversion, antisymmetry, Clifford algebra

## Abstract

The paper by Gopalan [(2020). *Acta Cryst.* A**76**, 318–327] presented an enumeration of the 41 physical quantity types in non-relativistic physics, in arbitrary dimensions, based on the formalism of Clifford algebra. Gopalan considered three antisymmetries: spatial inversion, 1, time reversal, 1′, and wedge reversion, 1^†^. A consideration of the set of all seven antisymmetries (1, 1′, 1^†^, 1′^†^, 1 ^†^, 1′, 1′^†^) leads to an extension of the results obtained by Gopalan. It is shown that there are 51 types of physical quantities with distinct symmetry properties in total.

## Abstract 2

It is shown that there are 51 types of physical quantities in arbitrary dimensions with distinct transformations by wedge reversion symmetry. In the paper by Gopalan [(2020). *Acta Cryst.* A**76**, 318–327] only 41 types were enumerated.

The paper by Gopalan (2020 ▸) presented an enumeration of the 41 types of physical quantities in non-relativistic physics in arbitrary dimensions within the formalism of Clifford algebra (Lounesto, 2009 ▸). This classification is based on three antisymmetries: spatial inversion, 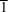 , time reversal, 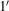 , and wedge reversion, 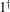 . [Note that, in Clifford algebra, spatial inversion 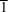 is termed (main) grade involution (Lounesto, 2009 ▸).] The 41 types of multivectors representing physical quantities were derived and presented in Table 1 of Gopalan (2020 ▸). Gopalan’s classification is an extension of the classification of three-dimensional vector-like physical quantities (Hlinka, 2014 ▸) to arbitrary dimensions.

The transformation of the physical properties represented by the principal multivectors S′, S, V′, V, B′, B, T′, T, or their combinations, under the antisymmetries 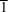 , 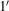 , 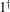 were considered by Gopalan (2020 ▸). S, V, B and T denote scalar, vector, bivector and trivector, respectively. The prime symbol ′ means invariance to time reversal, 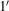 . There are three different outcomes of the operation, even (*e*), odd (*o*) or mixed (*m*). Mixed means that it is neither even nor odd, as explained in the short example in Table 1 ▸.

These outcomes for all physical properties are shown for each multivector type in Table 1 of Gopalan (2020 ▸) in columns titled ‘Action of 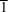 , 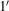 , 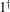 ’. The actions of the remaining antisymmetries 1′, 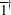 , 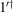 and 1′^†^ were not given in Table 1 of Gopalan (2020 ▸). [In Clifford algebra, the product of spatial inversion and wedge reversion, 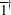 , is termed Clifford conjugation (Lounesto, 2009 ▸).] The consideration of all seven operations leads to new results, which are given here.

When a physical quantity is represented by a sum of two or more different principal multivector types then the action of at least four antisymmetries on this quantity gives mixed results. The analysis of the action of only three antisymmetries by Gopalan (2020 ▸) does not provide a unique solution.

Let *I*_1_ and *I*_2_ be two different antisymmetries (any out of the seven). If the action of both *I*_1_ and *I*_2_ on some multivector is mixed then the action of *I*_1_ · *I*_2_ on this multivector can be even, odd or mixed. Specifying the action of only three antisymmetries (especially 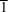 , 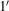 and 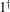 ) on a multivector, as was considered by Gopalan (2020 ▸), is not sufficient to obtain the result of the action of the remaining four antisymmetries; see a simplified example with three antisymmetries in Table 1 ▸. This has led to a clustering of different multivector types into one type in Table 1 of Gopalan (2020 ▸): the types numbered 16, 19, 22, 25, 28 and 31 should be separated into two types each and type 38 into five types. This gives in total ten new multivector types which were not given by Gopalan (2020 ▸), as shown in Table 2 ▸ for all seven antisymmetries. New labels for the X, Y, Z multivectors are proposed in Table 2 ▸ in a coherent notation, which uses four out of the eight principal multivectors. An extended version of Table 2 ▸ with grades and examples of multivectors is given in Table 3 ▸, which is the final table for these new results, with all 51 multivector types describing the action of all seven antisymmetries, given in the same layout as Table 1 of Gopalan (2020 ▸).

## Supplementary Material

Splitting of multivector types. DOI: 10.1107/S2053273323003303/ib5117sup1.pdf

## Figures and Tables

**Table 1:** An example of the action of 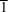 , 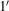 and 1′ antisymmetries on several multivectors The action of antisymmetries 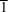 and 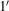 on S+V′ and S+V′+V gives mixed results, while the action of the product antisymmetry 1′ can be odd or mixed.

	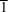	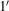	1′
S	+	−	−
	Even	Odd	Odd
			
V′	−	+	−
	Odd	Even	Odd
			
V	−	−	+
	Odd	Odd	Even
			
S+V′	+−	−+	−−
	Mixed	Mixed	Odd
			
S+V′+V	+−−	−+−	−−+
	Mixed	Mixed	Mixed

**Table 2:** Splitting of multivector types, with the left-hand side displaying the number, stabilizer subgroup, label and action of 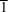 , 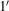 and 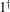 antisymmetries as given by Gopalan (2020 ▸), and the right-hand side displaying the number, stabilizer subgroup, label and action of all antisymmetries as presented in this work Considering the action of all antisymetries leads to splitting of multivector types. The last three rows describe the new labels of the X, Y and Z multivector types, without splitting. An extended version of this table with grades and examples of multivectors is available in the supporting information.

Work of Gopalan (2020 ▸)							This paper									
			Action of							Action of						
No.	SS	Label	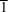	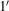	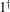		No.	SS	Label	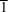	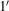	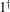	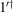	1′	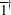	1′^†^
16	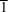	SB′(S′, B)	*e*	*m*	*m*	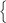	16*a*	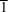	SB′	*e*	*m*	*m*	*o*	*m*	*m*	*o*
							16*b*		S′SB′B	*e*	*m*	*m*	*m*	*m*	*m*	*m*
																
19	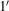	V′B′(S′, T′)	*m*	*e*	*m*	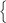	19*a*	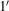	V′B′	*m*	*e*	*m*	*m*	*m*	*o*	*o*
							19*b*		S′V′B′T′	*m*	*e*	*m*	*m*	*m*	*m*	*m*
																
22	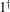	SV′(S′, V)	*m*	*m*	*e*	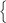	22*a*	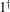	SV′	*m*	*m*	*e*	*m*	*o*	*m*	*o*
							22*b*		S′SV′V	*m*	*m*	*e*	*m*	*m*	*m*	*m*
																
25	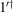	V′B(S′, T)	*m*	*m*	*m*	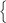	25*a*	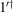	V′B	*m*	*m*	*m*	*e*	*o*	*o*	*m*
							25*b*		S′V′BT	*m*	*m*	*m*	*e*	*m*	*m*	*m*
																
28	1′	VB′(S′, T)	*m*	*m*	*m*	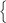	28*a*	1′	VB′	*m*	*m*	*m*	*o*	*e*	*o*	*m*
							28*b*		S′VB′T	*m*	*m*	*m*	*m*	*e*	*m*	*m*
																
31	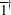	ST′(S′, T)	*m*	*m*	*m*	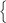	31*a*	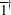	ST′	*m*	*m*	*m*	*o*	*o*	*e*	*m*
							31*b*		S′ST′T	*m*	*m*	*m*	*m*	*m*	*e*	*m*
																
38	1	W	*m*	*m*	*m*	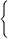	38*a*	1	SVB′T′	*m*	*m*	*m*	*o*	*m*	*m*	*m*
							38*b*		SV′BT′	*m*	*m*	*m*	*m*	*o*	*m*	*m*
							38*c*		V′VB′B	*m*	*m*	*m*	*m*	*m*	*o*	*m*
							38*d*		SV′B′T	*m*	*m*	*m*	*m*	*m*	*m*	*o*
							38*e*		S′SV′VB′BT′T	*m*	*m*	*m*	*m*	*m*	*m*	*m*
																
39	1	X	*m*	*m*	*o*	→	39	1	B′BT′T	*m*	*m*	*o*	*m*	*m*	*m*	*m*
																
40	1	Y	*m*	*o*	*m*	→	40	1	SVBT	*m*	*o*	*m*	*m*	*m*	*m*	*m*
																
41	1	Z	*o*	*m*	*m*	→	41	1	V′VT′T	*o*	*m*	*m*	*m*	*m*	*m*	*m*

**Table 3:** Classification of extended multivector types for physical properties using the same notation as in Table 1 of Gopalan (2020 ▸) The actions of all seven generalized inversions and grades are given explicitly. Entries in bold in columns 1 and 2 are the eight principal multivector types. Note that the last column contains sums (not products) of principal multivectors, but the ‘+’ signs are omitted.

				Action of								
New No.	Old No.	SS	Label	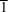	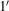	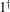	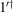	1′	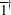	1′^†^	Grades	Multivectors (omitting ‘+’ signs)
**1**	**1**	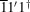	S′	*e*	*e*	*e*	*e*	*e*	*e*	*e*	4*g*	S′
												
**2**	**2**	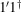	V′	*o*	*e*	*e*	*e*	*o*	*o*	*o*	4*g*+1	V′
3	3		S′V′	*m*	*e*	*e*	*e*	*m*	*m*	*m*	4*g*, 4*g*′+1	S′V′
												
**4**	**4**	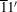	B′	*e*	*e*	*o*	*o*	*e*	*o*	*o*	4*g*+2	B′
5	5		S′B′	*e*	*e*	*m*	*m*	*e*	*m*	*m*	4*g*, 4*g*′+2	S′B′
												
**6**	**6**	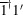	T′	*o*	*e*	*o*	*o*	*o*	*e*	*e*	4*g*+3	T′
7	7		S′T′	*m*	*e*	*m*	*m*	*m*	*e*	*e*	4*g*, 4*g*′+3	S′T′
												
**8**	**8**	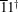	S	*e*	*o*	*e*	*o*	*o*	*e*	*o*	4*g*	S
9	9		S′S	*e*	*m*	*e*	*m*	*m*	*e*	*m*	4*g*, 4*g*′	S′S
												
**10**	**10**	1′ 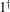	V	*o*	*o*	*e*	*o*	*e*	*o*	*e*	4*g*+1	V
11	11		S′V	*m*	*m*	*e*	*m*	*e*	*m*	*e*	4*g*, 4*g*′+1	S′V
												
**12**	**12**	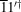	B	*e*	*o*	*o*	*e*	*o*	*o*	*e*	4*g*+2	B
13	13		S′B	*e*	*m*	*m*	*e*	*m*	*m*	*e*	4*g*, 4*g*′+2	S′B
												
**14**	**14**	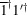	T	*o*	*o*	*o*	*e*	*e*	*e*	*o*	4*g*+3	T
15	15		S′T	*m*	*m*	*m*	*e*	*e*	*e*	*m*	4*g*, 4*g*′+3	S′T
												
16	16*a*	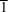	SB′	*e*	*m*	*m*	*o*	*m*	*m*	*o*	4*g*, 4*g*′+2	SB′
17	17		SB	*e*	*o*	*m*	*m*	*o*	*m*	*m*	4*g*, 4*g*′+2	SB
18	18		B′B	*e*	*m*	*o*	*m*	*m*	*o*	*m*	4*g*+2, 4*g*′+2	B′B
19	16*b*		S′SB′B	*e*	*m*	*m*	*m*	*m*	*m*	*m*	Three or four out of: 4*g*, 4*g*′, 4*g*′′+2, 4*g*′′′+2	SB′B, S′B′B, S′SB, S′SB′, S′SB′B
												
20	19*a*	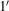	V′B′	*m*	*e*	*m*	*m*	*m*	*o*	*o*	4*g*+1, 4*g*′+2	V′B′
21	20		V′T′	*o*	*e*	*m*	*m*	*o*	*m*	*m*	4*g*+1, 4*g*′+3	V′T′
22	21		B′T′	*m*	*e*	*o*	*o*	*m*	*m*	*m*	4*g*+2, 4*g*′+3	B′T′
23	19*b*		S′V′B′T′	*m*	*e*	*m*	*m*	*m*	*m*	*m*	Three or four out of: 4*g*, 4*g*′+1, 4*g*′′+2, 4*g*′′′+3	V′B′T′, S′B′T′, S′V′T′, S′V′B′, S′V′B′T′
												
24	22*a*	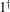	SV′	*m*	*m*	*e*	*m*	*o*	*m*	*o*	4*g*, 4*g*′+1	SV′
25	23		V′V	*o*	*m*	*e*	*m*	*m*	*o*	*m*	4*g*+1, 4*g*′+1	V′V
26	24		SV	*m*	*o*	*e*	*o*	*m*	*m*	*m*	4*g*, 4*g*′+1	SV
27	22*b*		S′SV′V	*m*	*m*	*e*	*m*	*m*	*m*	*m*	Three or four out of: 4*g*, 4*g*′, 4*g*′′+1, 4*g*′′′+1	SV′V, S′V′V, S′SV, S′SV′, S′SV′V
												
28	25*a*	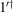	V′B	*m*	*m*	*m*	*e*	*o*	*o*	*m*	4*g*+1, 4*g*′+2	V′B
29	26		BT	*m*	*o*	*o*	*e*	*m*	*m*	*m*	4*g*+2, 4*g*′+3	BT
30	27		V′T	*o*	*m*	*m*	*e*	*m*	*m*	*o*	4*g*+1, 4*g*′+3	V′T
31	25*b*		S′V′BT	*m*	*m*	*m*	*e*	*m*	*m*	*m*	Three or four out of: 4*g*, 4*g*′+1, 4*g*′′+2, 4*g*′′′+3	V′BT, S′BT, S′V′T, S′V′B, S′V′BT
												
32	28*a*	1′	VB′	*m*	*m*	*m*	*o*	*e*	*o*	*m*	4*g*+1, 4*g*′+2	VB′
33	29		VT	*o*	*o*	*m*	*m*	*e*	*m*	*m*	4*g*+1, 4*g*′+3	VT
34	30		B′T	*m*	*m*	*o*	*m*	*e*	*m*	*o*	4*g*+2, 4*g*′+3	B′T
35	28*b*		S′VB′T	*m*	*m*	*m*	*m*	*e*	*m*	*m*	Three or four out of: 4*g*, 4*g*′+1, 4*g*′′+2, 4*g*′′′+3	VB′T, S′B′T, S′VT, S′VB′, S′VB′T
												
36	31*a*	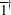	ST′	*m*	*m*	*m*	*o*	*o*	*e*	*m*	4*g*, 4*g*′+3	ST′
37	32		T′T	*o*	*m*	*o*	*m*	*m*	*e*	*m*	4*g*+3, 4*g*′+3	T′T
38	33		ST	*m*	*o*	*m*	*m*	*m*	*e*	*o*	4*g*, 4*g*′+3	ST
39	31*b*		S′ST′T	*m*	*m*	*m*	*m*	*m*	*e*	*m*	Three or four out of: 4*g*, 4*g*′, 4*g*′′+3, 4*g*′′′+3	ST′T, S′T′T, S′ST, S′ST′, S′ST′T
												
40	34	1′^†^	VB	*m*	*o*	*m*	*m*	*m*	*o*	*e*	4*g*+1, 4*g*′+2	VB
41	35		BT′	*m*	*m*	*o*	*m*	*o*	*m*	*e*	4*g*+2, 4*g*′+3	BT′
42	36		VT′	*o*	*m*	*m*	*o*	*m*	*m*	*e*	4*g*+1, 4*g*′+3	VT′
43	37		S′VBT′	*m*	*m*	*m*	*m*	*m*	*m*	*e*	Three or four out of: 4*g*, 4*g*′+1, 4*g*′′+2, 4*g*′′′+3	VBT′, S′BT′, S′VT′, S′VB, S′VBT′
												
44	38*a*	1	SVB′T′	*m*	*m*	*m*	*o*	*m*	*m*	*m*	Three or four out of: 4*g*, 4*g*′+1, 4*g*′′+2, 4*g*′′′+3	VB′T′, SB′T′, SVT′, SVB′, SVB′T′
45	38*b*		SV′BT′	*m*	*m*	*m*	*m*	*o*	*m*	*m*	Three or four out of: 4*g*, 4*g*′+1, 4*g*′′+2, 4*g*′′′+3	V′BT′, SBT′, SV′T′, SV′B, SV′BT′
46	38*c*		V′VB′B	*m*	*m*	*m*	*m*	*m*	*o*	*m*	Three or four out of: 4*g*+1, 4*g*′+1, 4*g*′′+2, 4*g*′′′+2	VB′B, V′B′B, V′VB, V′VB′, V′VB′B
47	38*d*		SV′B′T	*m*	*m*	*m*	*m*	*m*	*m*	*o*	Three or four out of: 4*g*, 4*g*′+1, 4*g*′′+2, 4*g*′′′+3	V′B′T, SB′T, SV′T, SV′B′, SV′B′T
48	39		B′BT′T	*m*	*m*	*o*	*m*	*m*	*m*	*m*	Three or four out of: 4*g*+2, 4*g*′+2, 4*g*′′+3, 4*g*′′′+3	BT′T, B′T′T, B′BT, B′BT′, B′BT′T
49	40		SVBT	*m*	*o*	*m*	*m*	*m*	*m*	*m*	Three or four out of: 4*g*, 4*g*′+1, 4*g*′′+2, 4*g*′′′+3	VBT, SBT, SVT, SVB, SVBT
50	41		V′VT′T	*o*	*m*	*m*	*m*	*m*	*m*	*m*	Three or four out of: 4*g*+1, 4*g*′+1, 4*g*′′+3, 4*g*′′′+3	VT′T, V′T′T, V′VT, V′VT′, V′VT′T
											
51	38*e*		S′SV′VB′BT′T	*m*	*m*	*m*	*m*	*m*	*m*	*m*	Varied	All other sums of: S′SV′VB′BT′T

## References

[bb1] Gopalan, V. (2020). *Acta Cryst.* A**76**, 318–327. 10.1107/S205327332000217X PMC7233017 32356782

[bb3] Hlinka, J. (2014). *Phys. Rev. Lett.* **113**, 165502. 10.1103/PhysRevLett.113.165502 25361266

[bb2] Lounesto, P. (2009). *Clifford Algebras and Spinors*, 2nd ed. Cambridge University Press.

