# Supplementary material for: A note on the wedge reversion antisymmetry operation and 51 types of physical quantities in arbitrary dimensions
Source: Acta Crystallogr A Found Adv. 2023 Jun 5;79(Pt 4):381–4. doi: 10.1107/S2053273323003303 (PMC10317133; doi:10.1107/S2053273323003303)
Supplement: Supplementary file 1 [file a-79-00381-sup1.pdf]

Acta Cryst A

FOUNDATIONS
ADVANCES

Volume 79 (2023)

Supporting information for article:

A note on the wedge reversion antisymmetry operation and 51 types of physical quantities in arbitrary dimensions

Piotr Fabrykiewicz

Supplementing Table. Splitting of multivector types. Columns from left to right denote: number, stabilizer subgroup, label, action of all antisymmetries, grades and examples of multivectors. Last rows describe new labels of X, Y and Z multivector types, without splitting. Note that the last column contains sums (not products) of principal multivectors, but the '+' signs are omitted.

				Action of								Multivectors
	No.	SS	Label	$\bar{1}$	$1'$	$1^\dagger$	$1'^\dagger$	$\bar{1}'$	$\bar{1}^\dagger$	$\bar{1}'^\dagger$	Grades	(omitting "+" signs)
Type	16	$\bar{1}$	SB′(S′, B)	*e*	*m*	*m*					$4g, 4g'+2$	B′S, SB′B, SS′B′, SS′BB′
splits to:	16*a*	$\bar{1}$	SB′	*e*	*m*	*m*	*o*	*m*	*m*	*o*	$4g, 4g'+2$	SB′
	16*b*		S′SB′B	*e*	*m*	*m*	*m*	*m*	*m*	*m*	3 or 4 out of: $4g, 4g', 4g''+2, 4g'''+2$	SB′B, S′B′B, S′SB, S′SB′, S′SB′B
Type	19	$1'$	V′B′(S′, T′)	*m*	*e*	*m*					All	B′V′, V′B′T′, S′V′B′, S′V′B′T′
splits to:	19*a*	$1'$	V′B′	*m*	*e*	*m*	*m*	*m*	*o*	*o*	$4g+1, 4g'+2$	V′B′
	19*b*		S′V′B′T′	*m*	*e*	*m*	*m*	*m*	*m*	*m*	3 or 4 out of: $4g, 4g'+1, 4g''+2, 4g'''+3$	V′B′T′, S′B′T′, S′V′T′, S′V′B′, S′V′B′T′
Type	22	$1^\dagger$	SV′(S′, V)	*m*	*m*	*e*					$4g, 4g'+1$	SV′, SVV′, SS′V′, SS′VV′
splits to:	22*a*	$1^\dagger$	SV′	*m*	*m*	*e*	*m*	*o*	*m*	*o*	$4g, 4g'+1$	SV′
	22*b*		S′SV′V	*m*	*m*	*e*	*m*	*m*	*m*	*m*	3 or 4 out of: $4g, 4g', 4g''+1, 4g'''+1$	SV′V, S′V′V, S′SV, S′SV′, S′SV′V
Type	25	$1'^\dagger$	V′B(S′, T)	*m*	*m*	*m*	*e*				Varied	V′B, V′BT, S′V′B, S′V′BT
splits to:	25*a*	$1'^\dagger$	V′B	*m*	*m*	*m*	*e*	*o*	*o*	*m*	$4g+1, 4g'+2$	V′B
	25*b*		S′V′BT	*m*	*m*	*m*	*e*	*m*	*m*	*m*	3 or 4 out of: $4g, 4g'+1, 4g''+2, 4g'''+3$	V′BT, S′BT, S′V′T, S′V′B, S′V′BT
Type	28	$\bar{1}'$	VB′(S′, T)	*m*	*m*	*m*		*e*			Varied	VB′, VB′T, VB′N, S′VB′T
splits to:	28*a*	$\bar{1}'$	VB′	*m*	*m*	*m*	*o*	*e*	*o*	*m*	$4g+1, 4g'+2$	VB′
	28*b*		S′VB′T	*m*	*m*	*m*	*m*	*e*	*m*	*m*	3 or 4 out of: $4g, 4g'+1, 4g''+2, 4g'''+3$	VB′T, S′B′T, S′VT, S′VB′, S′VB′T
Type	31	$\bar{1}^\dagger$	ST′(S′, T)	*m*	*m*	*m*			*e*		$4g, 4g'+3$	ST′, STT′, SS′T′, SS′TT′
splits to:	31*a*	$\bar{1}^\dagger$	ST′	*m*	*m*	*m*	*o*	*o*	*e*	*m*	$4g, 4g'+3$	ST′
	31*b*		S′ST′T	*m*	*m*	*m*	*m*	*m*	*e*	*m*	3 or 4 out of: $4g, 4g', 4g''+3, 4g'''+3$	ST′T, S′T′T, S′ST, S′ST′, S′ST′T
Type	38	1	W	*m*	*m*	*m*					Varied	e.g. S′VBT′ + any from Nos. 16 to 33
	38*a*		SVB′T′	*m*	*m*	*m*	*o*	*m*	*m*	*m*	3 or 4 out of: $4g, 4g'+1, 4g''+2, 4g'''+3$	VB′T′, SB′T′, SVT′, SVB′, SVB′T′
	38*b*		SV′BT′	*m*	*m*	*m*	*m*	*o*	*m*	*m*	3 or 4 out of: $4g, 4g'+1, 4g''+2, 4g'''+3$	V′BT′, SBT′, SV′T′, SV′B, SV′BT′
splits to:	38*c*	1	V′VB′B	*m*	*m*	*m*	*m*	*m*	*o*	*m*	3 or 4 out of: $4g+1, 4g'+1, 4g''+2, 4g'''+2$	VB′B, V′B′B, V′VB, V′VB′, V′VB′B
	38*d*		SV′B′T	*m*	*m*	*m*	*m*	*m*	*m*	*o*	3 or 4 out of: $4g, 4g'+1, 4g''+2, 4g'''+3$	V′B′T, SB′T, SV′T, SV′B′, SV′B′T
	38*e*		S′SV′VB′BT′T	*m*	*m*	*m*	*m*	*m*	*m*	*m*	Varied	All other sums of: S′SV′VB′BT′T
Type	39	1	X	*m*	*m*	*o*					Varied	e.g. BT′ TT′ B′T BT B′T′ BB′ T T′ B B′
changes to:	39	1	B′BT′T	*m*	*m*	*o*	*m*	*m*	*m*	*m*	3 or 4 out of: $4g+2, 4g'+2, 4g''+3, 4g'''+3$	BT′T, B′T′T, B′BT, B′BT′, B′BT′T
Type	40	1	Y	*m*	*o*	*m*					Varied	e.g. VB ST VT BT SV SB S
changes to:	40	1	SVBT	*m*	*o*	*m*	*m*	*m*	*m*	*m*	3 or 4 out of: $4g, 4g'+1, 4g''+2, 4g'''+3$	VBT, SBT, SVT, SVB, SVBT
Type	41	1	Z	*o*	*m*	*m*					Varied	e.g. VT′ TT′ VT V′T VV′ V′T′ V V′ T T′
changes to:	41	1	V′VT′T	*o*	*m*	*m*	*m*	*m*	*m*	*m*	3 or 4 out of: $4g+1, 4g'+1, 4g''+3, 4g'''+3$	VT′T, V′T′T, V′VT, V′VT′, V′VT′T
